# Effect of Various Types of Intermittent Fasting (IF) on Weight Loss and Improvement of Diabetic Parameters in Human

**DOI:** 10.1007/s13668-021-00353-5

**Published:** 2021-04-07

**Authors:** Karolina Nowosad, Monika Sujka

**Affiliations:** Department of Analysis and Evaluation of Food Quality, University of Life Sciences in Lublin, Skromna 8, 20-704 Lublin, Poland

**Keywords:** Anthropometric parameters, Intermittent fasting, Obesity, Fasting glucose, Fasting insulin, HOMA-IR

## Abstract

**Purpose of Review:**

A number of recent studies have suggested that intermittent fasting is as effective as traditional calorie restriction (CR) for weight loss and for cardioprotection. However, it is still unclear whether IF improves diabetes risk indicators as does CR. This review provides an overview of various patterns of intermittent fasting and shows the effect of intermittent fasting on human anthropometric such as excess body weight and biochemical parameters for example high glucose and fasting insulin, which are risk factors for diabetes.

**Recent Findings:**

There is a growing body of evidence pointing to the benefits of intermittent fasting for glucose and insulin homeostasis, but this should be confirmed by further studies in population groups with (or at high risk) type II diabetes and insulin resistance. Long-term studies are also needed that could reveal potential negative health effects that some studies report.

**Summary:**

Eleven studies in overweight/obese adult people that included changes in weight, body composition, and diabetic parameters (fasting glucose, fasting insulin, HbA1c concentration, and HOMA-IR index) were published between 2012 and 2020. Seven studies concerning the effects of alternate day fasting (ADF) on weight loss and diabetic parameters were analyzed. All of them have shown the effects of ADF on weight loss and slight improvement in diabetic parameters. For time-restricted feeding (TRF), a significant improvement in the HOMA-IR index was observed in 2 studies. One study saw an increase in fasting glucose. An analysis of 2 studies using a complete alternate day fasting (CADF) was performed. One study showed decrease in fasting glucose and insulin, and in one a decrease in glycosylated hemoglobin (HbA1c) was observed.

**Conclusion:**

Different types of intermittent fasting reduce body weight and reduce diabetes parameters such as fasting glucose, fasting insulin, HOMA-IR index, and glycated hemoglobin (HbA1c).

## Introduction

The number of overweight or obese people is constantly increasing. Obesity is one of the factors in the development of insulin resistance and diabetes. A weight loss of 1 kg reduces the risk of diabetes by as much as 16% [1]. To reduce weight and improve body composition (fat, muscle, and water contents), it is necessary to introduce physical activity and a diet with a negative calorie balance. There are a number of weight loss diets that allow to lose body fat and reduce the risk of diseases associated with excessive body weight [1]. One of the alternative methods of reducing kilocalories (kcal) intake is intermittent fasting [2]. Intermittent fasting (IF) is a broad term encompassing various programs that manipulate meal time, using short-term fast to improve body composition and overall health [3]. This method consists in refraining from eating for a specific period of time, e.g., 16 h, and then eating meals in so-called feeding window, which lasts, e.g., 8 h. IF can be modified — periods of fasting or “eating window” were lengthened or shortened [2]. An example of such a modification is alternating eating 1 day and then fasting the next day. On the day of fasting, one meal (most often consumed at lunch) is usually eaten, which does not exceed 25% of the daily caloric demand [4]. Another example of IF modification is the introduction of 1 or 2 days fasting per week. During the fast, one can completely eliminate food or reduce calories to a minimum [5] (Tables 1 and 2).

**Table 1:** Types of intermittent fasting [6], with permission

Complete alternate day fasting	Alternating fasting days (without consuming high energy food and drinks) with eating days (eating and drinking ad libitum)
Alternate day fasting	It allows consumption from 20 to 25% of energy demand on scheduled fasting days. This scheme forms the basis of the popular 5:2 diet, which is associated with severe energy restriction for 2 uninterrupted days a week and eating ad libitum for the remaining 5 days.
Time-restricted feeding	Eating meals with a certain energy value during the “food window,” which lasts several hours. The most common modification is eating for 8 h, followed by fasting for the next 16 h. The hours of fasting and eating can be shortened and extended.
Religious fasting	A wide range of fasts is undertaken for religious or spiritual purposes

**Table 2:** Sample schedule of food intake during various modifications of IF [3], with permission

Modification of IF	Day 1	Day 2	Day 3	Day 4	Day 5	Day 6	Day 7
Alternate day fasting (ADF)	Ad libitum	25% kcal	Ad libitum	25% kcal	Ad libitum	25% kcal	Ad libitum
Time-restricted feeding (TRF)	16–20 h of fasting, 4–8 h of feeding	16–20 h of fasting, 4–8 h of feeding	16–20 h of fasting, 4–8 h of feeding	16–20 h of fasting, 4–8 h of feeding	16–20 h of fasting, 4–8 h of feeding	16–20 h of fasting, 4–8 h of feeding	16–20 h of fasting, 4–8 h of feeding
Complete alternate day fasting (CADF)	Ad libitum	Ad libitum	Ad libitum	Ad libitum/24-h fast	Ad libitum	Ad libitum	24-h fast

Glucose and lipid homeostasis can be improved by a slight weight loss, which, according to current dietary recommendations, is most often achieved through moderate continuous (daily) energy reduction (CER). Regardless of weight loss, metabolic regulation of glucose and lipids can also be affected by other dietary manipulations, including changes in meal time [7] as well as sudden changes in energy status, such as fasting [8]. Most studies on IF have considered whether IF could be a potential strategy to lose weight and improve diabetic parameters in human [9]. Several modifications of IF have gained popularity as they offer impressive health benefits. However, it is unlikely that all IF schemes lead to the same physiological changes because of their different fasting and feeding patterns [3].

A number of recent studies have suggested that IF is also effective, as is traditional calorie restriction (CR) for weight loss and for improve diabetic parameters. However, it is still unclear whether IF improves diabetes risk indicators as does CR. This review provides an overview of various patterns of IF and shows the effect of IF on human anthropometric and biochemical parameters (Table 3). The review focuses mainly on human research in recent years. The main aim of this article is to present the effects of IF on weight loss, fasting glucose, fasting insulin, and insulin sensitivity in overweight and obese adults.

**Table 3:** Effect of IF on anthropometric and biochemical characteristics

Author	Type of IF	Purpose	Participants	Intervention	Weight and body composition (kg or %)			Diabetic parameters			
					Body weight	Fat mass	Lean mass	Fasting glucose	Fasting insulin	HOMA-IR	HbA1c
Sutton et al. [10^•^]	TRF	The impact of TRF on diabetic parameters	One hundred thirty men in pre-diabetes condition, 56±9 years old; BMI 32.2±4.4 kg/m^2^, fasting glucose 102±9 mg/dL, fasting insulin 25.1±14.5 mU/L	Five-week study, randomized, cross-over, isocaloric, and eucaloric TRF: 6-h daily food period, dinner before 15:00 or 12-h food period	TRF: ↓1.4±1.3 kg to↓1.0±1.1 kg; p=0.12	Not studied	Not studied	TRF: no effect (Δ=2±2 mg/dL; p=0.49)	TRF:↓3.4±1.6 mU/L; p=0.05	Not studied	Not studied
Gabel et al. [11^••^]	TRF	The effect of TRF nutrition for 8 h on body weight and risk factors for metabolic diseases in obese adults	Forty-six obese people, BMI 30–45 kg/m^2^, age 25–65, no diabetes, study group (n=23), control group (n=23)	A pilot study: 8-h nutritional intervention (feeding ad libitum between 10:00 and 18:00, fasting from 18:00 to 10:00) for 12 weeks. Weight loss and other results were compared with the matched control group	TRF: ↓3±3 kg Control: no change; *p*<0.001	TRF: ↓2±2 kg Control: no change (before 37±2 kg; after 37±2 kg); *p*<0.23	TRF: no change (before 50±2 kg; after 50±2 kg) Control: no change (before 53±2 kg; after 53±2 kg); *p*<0.12	TRF: before: 79±4 mg/dL, after: 82±2 mg/dL Control: no change (before and after: 87±2 mg/dL); *p*<0.77	TRF: before: 8.3±1 uIU/mL, after: 5.7±0.7 uIU/mL Control: before: 9.2±1.4 uIU/mL; after 10.3±1.9 uIU/mL; *p*<0.16	TRF: before: 1.6±0.2, after: 1.0±0.2 Control: before: 2.0±0.2; after 2.2±0.4; *p*<0.21	Not studied
Arnason et al. [12]	TRF	The effect of IF on biochemical parameters in people with type II diabetes	Ten participants with a confirmed diagnosis of T2DM with a BMI of 36.90 kg/m^2^, age 44-62, Participants were taking metformin	A pilot study: three-phase observational study (phase 1: standard diet, phase 2: TRF, phase 3: standard diet)	Mean difference phase 1 to 2: ↓1.4 kg (p=0.009) BMI: ↓0.52 kg/m^2^ (p=0.01) Mean difference phase 2 to 3: ↑0,28 kg (p=1.0) BMI: ↑0.1 kg/m^2^, (p=1.0)	Not studied	Not studied	% change from phase 1 to 2: ↓6.10% (p=0.002) % change from phase 2 to 3: ↑5.2% (p=0.003)	Not studied	Mean difference phase 1 to 2: ↓0.46 (p=1.0) Mean difference phase 2 to 3: ↑0,11 (p=1.0)	Not Studied
Catenacci et al. [13]	ADF	The comparison of the effects of the ADF diet on changes in body weight and insulin sensitivity index (Si) with changes in a standard weight loss diet with moderate daily caloric restriction (CR)	Fourteen obese adults with a BMI 30 to 52 kg/m^2^, aged 18–55, nonsmoker	Randomly assigning 14 people to the group with the ADF diet or 14 people to the CR diet (−400 kcal/day, n=2) for 8 weeks	ADF: ↓8.2±0.9 kg CR: ↓7.1±1.0 kg *p*<0.001	ADF: ↓3.7±0.5 kg CR: ↓3.7±0.5 kg *p*<0.001	ADF: ↓3.2±0.6 kg CR: ↓2.6±0.6 kg *p*<0.001	ADF: ↓6.0±2.1 mg/dL, p=0.01 CR: ↓3.3±2.3 mg/dL, p=0.166	ADF: ↓3.0±2.3μU/mL, p=0.207 CR: ↓0.2±2.4 μU/mL, p=0.945	Not studied	Not studied
Carter et al. [14]	ADF	The effects of intermittent energy restriction (IER) compared to continuous energy restriction (CER) on glycated hemoglobin A1c (HbA1c)	Sixty-three overweight or obese participants age ≥18 years, BMI 35.2±5 kg/m^2^ with T2DM (HbA1c 57 mmol/mol)	A pragmatic pilot trial: Group 1: 2-day calorie restriction (1670–2500 kJ/day) and 5-day eating with old eating habits Group 2: moderate diet with normal energy supply (5000–6500 kJ/day) The study lasted 12 weeks	IF: 100±20 kg to 92±14 kg CER: 102±17 kg to 94±13 kg p=0.7	IF: ↓3.8±2.7 kg CER: ↓4.0±3.2 kg *p*<0.001	IF: ↓2.2±1.9 kg CER: ↓1.1±2.1 kg *p*<0.001	Not studied	Not studied	Not studied	IF: ↓0.8±1.0% CER: ↓0.6±0.8% p=0.3
Harvie et al. [15]	ADF	The comparison of two intermittent energy and carbohydrate restriction (IECR) dietary regimens with a diet that allowed eating protein and fat ad libitum (IECR + PF)	Thirty-seven women with a BMI 29.6±4.1 and aged 45.6±8.3 kg	Randomized trial, those on the IECR diet consumed an average of 2500–2717 kJ/day or were on a 25% reduced calorie diet with reduced carbohydrate content (<40g carbohydrate/day) The second group consumed protein and fat ad libitum and<40 g of carbohydrates/day. Forty people were the control group on a reduced calorie diet (DER)	IECR: ↓5 kg IECR+PF: ↓5 kg DER: ↓5 kg p=0.1	IECR: ↓3.7 kg IECR+PF: ↓3.7 kg DER: ↓2 kg p=0.04	IECR: ↓2 kg IECR+PF: ↓2 kg DER: ↓2 kg p=0.288	IECR: no change IECR+PF: no change DER: no change	IECR: ↓9 pmol/L p=0.017 IECR+PF: ↓8 pmol/L p=0.176 DER: no change	IECR: ↓0.4 p=0.02 IECR+PF: ↓0.3 p=0.21 DER: no change	IECR: no change IECR+PF: no change DER: no change
Trepanowski et al. [16]	ADF	Effects of alternate-day fasting (ADF) or daily calorie restriction (CR) on body composition and diabetic parameters.	Seventy-nine men and women (ADF n=25; CR n=29; control n=25); overweight or obese, aged 18–65	Participants were allocated to three groups: ADF, CR, and control. The study included a 4-week baseline period; 24-week intervention period aimed at weight loss; 24-week weight maintenance period	Not studied	ADF: ↓10±2% CR: ↓15±3% (compared to the control group) *p*<0.01	ADF: ↓1.2±0.4kg CR: ↓1.8±0.8kg Control: ↓0.3±0.4kg *p*<0.01	ADF: no change CR: ↓5.2**±**0.1 mg/mL Control: ↓5±0.6 mg/mL *p*<0.01	ADF: ↓42±12% CR: ↓23±10% *p*<0.05	ADF: ↓45±13% CR: ↓18±11% Control: ↑14±17% *p*<0.05	Not studied
Bhutani et al. [17]	ADF	Compare the effects of ADF in conjunction with exercise with ADF intervention alone on body composition and diabetes parameters	Eighty-three participants age 25–65 years; BMI between 30 and 39.9 kg/m^2^	Participants were randomized to 1 of 4 groups for 12 weeks: (1) combination (ADF plus endurance exercise), (2) ADF, (3) exercise, or (4) control	ADF + exercise ↓6±4kg ADF: ↓3±1kg Exercise ↓1±0kg control: ↓0±0kg *p*<0.001	ADF + exercise ↓5±1kg ADF: ↓2±1kg Exercise ↓1±0kg control: ↓0±1kg *p*<0.001	ADF + exercise ↓0±1kg ADF: ↓1±1kg Exercise ↓1±0kg control: ↓1±1kg p=0.527	ADF + exercise ↓2±4 mg/dL ADF: ↓3±2 mg/dL Exercise ↓1±2 mg/dL control: ↑2±4 mg/dL p=0.461	ADF + exercise ↓21±15 μIU/mL ADF: ↓7±6 μIU/mL Exercise ↓0±8 μIU/mL control: ↓16±9 μIU/mL p=0.559	ADF + exercise ↓0±17 ADF: ↓0±7 Exercise ↓0±10 control: ↓0±11 p=0.589	Not studied
Harvie et al. [18]	ADF	Compare the effectiveness of ADF and caloric restriction (CER) on weight loss and insulin sensitivity	One hundred seven pre-menopausal women aged 30–45 years, with overweight and obesity, BMI 24–40 kg/m^2^	The women were randomly assigned to the following groups: IER (25% energy restriction 2 days a week, ~2710 kJ/day) and CER (daily calorie restriction ~6276 kJ/day for 7 days)	IER: ↓6.4 (7.9 to 4.8) kg CER: ↓5.6 (6.9 to 4.4) kg p=0.26	IER: ↓4.5 (4.9 to 4.1) kg CER: ↓3.6 (4 to 3.2) kg p=0.34	IER: ↓1.2 (1.4 to 1.1) kg CER: ↓0.8 (1 to 0.6) kg p=0.21	IER: ↓0.1 (0.1 to 0.1) mmol/L CER: ↓0.1 (0.1 to 0) mmol/L p=0.34	IER: ↓**2**.1 (1.8 to 2.4) μU/mL CER: ↓1.1 (1 to 1.2) μU/mL p=0.04	IER: ↓0.4 (0.4 to 0.5) CER: ↓0.3 (0.2 to 0.2) p=0.04	Not studied
Antoni et al. [8]	CADF, ADF	Comparison of an isocaloric diet with 75% (ADF) and 100% (CADF) caloric restriction for glucose metabolism	Fourteen overweight or obese participants, aged 18–60 years	The study was a three-way, randomi**z**ed, cross-over study. Participants were three types of 1-day diets at random: isocaloric, TRF, and ADF diets	Not studied	Not studied	Not studied	Isoenergetic diet: 4.7±0.1 mmol/L CADF: 4.3±0.2 mmol/L ADF: 4.4±0.1 mmol/L	Isoenergetic diet: 89.7±9.2 pmol/L CADF: 74.0±12.4 pmol/L ADF: 69.1±8.0 pmol/L	Not studied	Not studied
Lichtash et al. [19•]	CADF	The effect of IF and the ketogenic diet on the improvement of HbA1c in a patient with type II diabetes	A 57-year-old woman with 15 years of type II diabetes, HbA1c 9.3%, BMI 23.2 kg/m^2^	Use of a ketogenic diet combined with IF for 14 months	IF: ↓1.8 kg *p*<0.05	Not studied	Not studied	Not studied	Not studied	Not studied	IF: ↓3.5% *p*<0.05

## Search Strategy and Selection Criteria

### Eligibility Criteria

This review seeks to answer the research question “How do different types of intermittent fasting affect diabetes parameters in overweight or obese people?” The inclusion and exclusion criteria and search strategy were developed according to the PICO framework.

### Inclusion Criteria

- Population: obese or overweight people with a BMI above 25.0 or people with normal body weight with type II diabetes, aged >18 years, women and men.
- Problem: excess body weight, abnormal fasting glucose or insulin levels, HOMA-IR, or glycosylated hemoglobin.
- Intervention: use of different types of intermittent fasting (TRF, ADF, CADF).
- Result: changes in body composition (weight loss, fat, and lean mass loss), change in biochemical parameters (fasting glucose, fasting insulin, HOMA-IR index, glycated hemoglobin).
- Type of study: randomized controlled trials, cross-over, pilot studies, case studies, published between 2005 and 2020, written in English.

### Exclusion Criteria

- Population: children aged <18 years, people with normal body weight without type II diabetes.
- Problem: no changes in biochemical parameters, alternating several types of intermittent fasting.
- Intervention: using of intermittent fasting for a short time, e.g., 1 day.
- Result: does not directly measure the effects of an intervention.
- Type of research: review articles, opinion articles, editorials, letters, news reports, policy or legislative literature; animal research; published before 2005 or after 2020, written in a language other than English.

Two databases, PubMed and Scopus, were searched in September 2020 to find research and review articles concerning an impact of intermittent fasting on human health. The following phrases were searched: “post IF,” “intermittent fasting,” “IF human.” Then, in order to narrow the search, the following keywords were used: “impact of IF,” “influence IF on human health,” “IF diabetic parameters,” “IF glucose,” “intermittent fasting glucose,” “IF body mass,” “intermittent fasting body mass,” “IF lose weight,” “intermittent fasting insulin.” Then we searched for articles describing the impact of a specific type of periodic fasting (ADF, TRF, CADF) on human anthropometric and biochemical parameters: “ADF human health,” “ADF glucose,” “ADF body mass,” “ADF weight,” “ADF insulin,” “ADF HOMA-IR,” “ADF HbA1c,” “TRF human health,” “TRF glucose,” “TRF body mass,” “TRF weight,” “TRF insulin,” “TRF HOMA-IR,” “TRF HbA1c” and “CADF human health,” “CADF glucose,” “CADF body mass,” “CADF weight,” “CADF insulin,” “CADF HOMA-IR,” “CADF HbA1c.” The articles were examined based on the title and summary. When the abstract was not available or the information was insufficient to decide on inclusion in the review article, the full text was read. Then the following data was extracted: size and details of the research or the control group, duration, nutrition strategy, and measure of results.

## Results and Discussion

Eleven studies were published between 2005 and 2020. We analyzed three studies describing the effects of TRF on weight loss and diabetics parameters. All studies have shown the effects of TRF on weight loss, but in one study, this effect is not statistically significant. One study additionally investigated the effects of TRF on fat loss. TRF also reduced fasting glucose (in 1 case) and fasting insulin (in 2 cases). In two studies, time-restricted feeding had an effect on reducing HOMA-IR. The effect of TRF on HbA1c levels has not been studied in any of the articles. For ADF, significant weight loss was observed in 5 studies and it was caused by a loss of body fat. Four studies showed reduction in fasting glucose and one study in HbA1c. In six studies, ADF had an effect on fasting insulin levels and in four studies on HOMA-IR index. In 2 studies in which post-CADF was used, a decrease in fasting glucose and fasting insulin was observed. This had the effect of reducing the fasting glucose and insulin (which was tested in one of the studies). HbA1c decreased in 1 case. Detailed information about the research can be found in the following paragraphs.

Sutton et al. [10^•^] conducted a 5-week RCT study among 130 men with pre-diabetes. The participants were assigned to the TRF eating schedule, which consisted of a 6-h eating window and eating the last meal at 3 p.m., and to the control group, which had an extended eating window to 12 h. Men started the eating window at 8.00/8.30. Study participants ate only meals prepared by staff and received the amount of calories needed to maintain their weight. After 5 weeks of TRF, participants had an improvement in insulin sensitivity relative to baseline. The use of TRF did not affect the energy expenditure among the respondents. Probably the insignificant difference in body weight between the control group and the test group was caused by the reduction of glycogen levels and the accompanying water loss.

Gabel et al. [11^••^] also introduced an 8-h eating window for 23 obese people. In this pilot study, participants ate meals ad libitum in the eating window for 12 weeks. Another 23 people (randomly selected) constituted the control group, whose members were asked to maintain their weight throughout the study and not to change their eating habits or physical activity. The participants were tested for body composition, glycemic, and lipid parameters. After 12 weeks of using the TRF dietary regimen, weight loss and improvement in blood pressure were observed. There were no statistically significant differences between the groups in the case of fat mass and lean mass. TRF did not significantly improve fasting glucose but caused a decrease of fasting insulin levels and thereby improved HOMA-IR.

Arnason et al. [12] investigated the short-term biochemical effects of intermittent fasting (TRF) in 10 adults with type 2 diabetes (T2DM) taking metformin. The pilot study consisted of three phases (baseline — 2 weeks, intervention — 2 weeks, observation — 2 weeks), followed by biochemical and anthropometric measurements, and the level of participants’ activity. Patients had the choice of using intermittent fasting in the morning, afternoon, or evening; however, all participants preferred to start their meals in the afternoon. Short-term intermittent fasting resulted in significant weight loss (−1.395 kg), BMI (−0.517), and at-target morning glucose (SMBG). SMBG (self-monitoring of blood glucose) is an important component of self-management in diabetes and enables it people with diabetes make the daily decisions necessary to achieve and maintain proper control glycemia [12]. Increasing the duration of fasting improved fasting glucose values. Neither insulin resistance (HOMA-IR) nor markers of inflammation (C-reactive protein) did not normalize during intermittent fasting. During phase 3 (standard diet), there was a slight increase in weight and BMI of the study participants, which also resulted in an increase in fasting glucose (by 5.2%). Intermittent fasting reduced post-prandial hyperglycemia (39.4% vs 47.4% of the baseline value). The weight loss was due to a reduced caloric intake (18% from baseline), which resulted in improved participants’ diabetes parameters.

The randomized pilot study by Catenacci et al. [13] concerned the effect of ADF on body weight, body composition, and the insulin sensitivity index (Si). A total 26 obese subjects participated in the study, with 14 subjects assigned to an intermittent fasting diet, while the remaining 12 subjects were the controls on a standard low-calorie diet. The study lasted 8 weeks. For ADF, participants achieved a 376 kcal greater deficit than participants in the control group, but this significantly affects weight, body composition, and insulin sensitivity index.

Carter et al. [14] made similar conclusions. Their study involved 63 overweight and obese people who were assigned ADF and a standard reduction diet with a caloric value of 5000–6500 kJ/day. ADF consisted of a 2-day caloric restriction of 1670–2500 kJ/day and a 5-day ad libitum diet for 12 weeks. There were no significant differences between the two groups in terms of body weight, body composition, and improvement in HbA1c.

Improvement in insulin sensitivity was observed in randomized trial by Harvie et al. [15]. Two dietary regimens were tested in their study: intermittent fasting with energy and carbohydrate restriction (IECR) and intermittent fasting, which allowed ad libitum protein and fat (IECR + PF), and the results were compared to a daily calorie-restricted diet. The intermittent fasting regimen included a 25% calorie reduction 2 days a week. The study involved 115 overweight women who reported breast cancer in a family history. A reduction in the fasting indicators of insulin resistance (fasting glucose, fasting insulin, HOMA-IR index) was observed in both groups. A reduction in body fat percentage was observed for both groups using intermittent fasting (IECR: average — 3.7 kg; IECR + PF: average — 3.7 kg; DER: average — 2.0 kg).

In the RCT study by Trepanowski et al. [16], participants were randomized to an ADF diet, a caloric restriction (CR) group, and a control group. Both ADF and CR significantly improved participants’ anthropometric parameters. In the group on ADF, no changes in fasting glucose levels were observed, while improvements in fasting insulin and the associated HOMA-IR index were observed.

Bhutani et al. [17] investigated the effect of ADF in combination with exercise on changes in body composition and biochemical parameters in the randomized study. For this purpose, obese people were assigned to 4 groups: ADF with exercise, ADF, exercise, and a control group. Body weight decreased in the ADF with exercise, ADF, and exercise only groups by 6 ± 4 kg, 3 ± 1 kg, and 1 ± 0 kg, respectively, at p <0.005. ADF and exercise also improved diabetes parameters (fasting insulin levels and HOMA-IR index). Weight loss was associated with an improvement in cell sensitivity to insulin.

In the randomized study by Harvie et al. [18], the effects of intermittent fasting (ADF) were compared with caloric restriction in terms of weight loss and improvement in insulin sensitivity. The study involved 107 women with BMI>30 kg/m^2^. Both ADF and caloric restriction were shown to decrease body weight by −6.4 (−7.9 to −4.8) kg and −5.6 (−6.9 to −4.4) kg, respectively. The improvement in fasting insulin levels was modest in both group, but was greater in the ADF group. Both intermittent fasting and daily caloric reduction have been shown to be an effective method of losing weight and improving diabetic parameters.

In the randomized, cross-over study by Antoni et al. [8], participants randomly followed three 1-day diets: an isocaloric diet, 75% calorie restriction (ADF), and 100% calorie restriction (CADF). The study compared the effects of an isocaloric diet and intermittent fasting on glucose metabolism. The study shows the significant effect of CADF and ADF in reducing fasting glucose and insulin levels. The lowest fasting glucose and insulin levels were obtained with CADF, and the highest with an isocaloric diet.

The effect of intermittent fasting in combination with a ketogenic diet on HbA1c in a healthy body weight woman with type II diabetes was investigated in a case study. The ketogenic diet consisted of the following macronutrient composition: 5% carbohydrates, 15% protein, and 80% fat. The patient had not previously seen any improvement in glycaemia following the standard dietary approach (low glycemic index diet with calorie restriction). Intermittent fasting was introduced for 24 h three times a week, then 42 h three times a week, then 42 h twice a week, and 16 h a week. After 8 months, the IF was reduced to 16 h a day, with 24 h three times a month, and metformin was reintroduced. After 14 months, HbA1c reached 5.8%, and the body mass index slightly changed [19•]. When selecting the study, the time of eating meals was taken into account in accordance with the idea of intermittent fasting, because the ketogenic diet determines the type of food that should be eaten (it shows what nutrients should consist of a meal), but does not contain indications regarding the time of meals (i.e., when the meal should be eaten).

## Conclusions

Pre-clinical and clinical studies have shown that intermittent fasting has a wide range of benefits for many diseases, including obesity, diabetes, and insulin resistance. The results of human studies, in which various indicators of health are measured at the beginning and after periods of IF of 2–6 months or longer, suggest that IF may protect against diabetes and obesity.

Recent studies have produced promising results that strongly support further clinical trials in patients with chronic age-related disorders and obesity. An important clinical and scientific question is whether adopting a regular, intermittent fasting regimen is a viable and sustainable strategy that can be used in health promotion. In addition, adequate clinical trials are needed to see if intermittent fasting can supplement or replace energy restriction, and if so, can facilitate long-term improvement in metabolism and weight control. This review suggests that intermittent fasting regimens may be a promising approach to losing weight and improving blood chemistry in people who can safely tolerate interruptions or eat very little during certain hours of the day, night, or days of the week.
